# Real‐world safety and effectiveness of pembrolizumab in Japanese patients with radically unresectable melanoma: An all‐case postmarketing surveillance in Japan

**DOI:** 10.1111/1346-8138.16518

**Published:** 2022-07-27

**Authors:** Naoya Yamazaki, Akiko Shimizu, Masahiko Ozaki, Masahiro Hamada, Noriko Takeuchi, Yuichiro Ito, Shinichiroh Maekawa

**Affiliations:** ^1^ National Cancer Center Hospital Tokyo Japan; ^2^ Oncology Medical Affairs, MSD K.K. Tokyo Japan; ^3^ Japan Pharmacovigilance, MSD K.K. Tokyo Japan

**Keywords:** Japan, melanoma, pembrolizumab, postmarketing surveillance, safety and effectiveness

## Abstract

This all‐case postmarketing surveillance (PMS) survey (101 centers; February 15, 2017, to March 3, 2020) captured factors that impact the safety and effectiveness of newly initiated pembrolizumab monotherapy for the treatment of radically unresectable melanoma in Japan. Eligible patients were enrolled both retrospectively and prospectively, and followed up at 1, 3, 6, and 12 months. Safety assessments included treatment‐related adverse events (TRAEs), adverse events of special interest (AEOSIs) from the Japanese Risk Management Plan (J‐AEOSIs), and J‐AEOSIs related to pembrolizumab. Effectiveness assessments included objective response rate (ORR; complete response/partial response) and disease control rate (DCR) according to the RECIST criteria. Overall, 294 and 236 patients comprised the safety and effectiveness (RECIST) assessment sets, respectively. Median (range) age of the patients was 70 (22–94) years, and the majority (60.4%) received pembrolizumab as first‐line therapy. The most common type of melanoma was cutaneous (41.5%), followed by mucosal (29.3%), acral (24.8%), and unknown (4.4%). Overall, 45.2% and 24.8% of patients experienced TRAEs and AEOSIs, respectively. In total, 24.8% and 9.2% of patients experienced any‐grade and grade ≥3 pembrolizumab‐related AEOSIs, respectively. The most common grade ≥3 pembrolizumab‐related AEOSIs were endocrine disorders and liver dysfunction (2.4% each), followed by colitis/severe diarrhea (2.0%), interstitial lung disease (1%), and type 1 diabetes (0.7%). No grade 5 J‐AEOSIs were observed. ORR was 16.5% at the 1‐year follow‐up: mucosal melanoma (20%), acral melanoma (10%), and cutaneous melanoma (17.5%). ORR was higher among patients who did not receive versus those who did receive previous systemic therapy across all three melanoma types. DCR was 52.1% at the 1‐year follow‐up: cutaneous melanoma (57.3%), acral melanoma (51.7%), and mucosal melanoma (43.1%). This all‐case PMS survey confirmed the real‐world safety and effectiveness of pembrolizumab monotherapy for the treatment of radically unresectable melanoma in Japan.

## 1 INTRODUCTION

According to the 2020 GLOBOCAN estimates, melanoma of the skin is among the 15 most common cancers in men, with 324 635 new cases and 57 043 new deaths reported worldwide.^1^ Melanoma is the third most common type of skin cancer in Japan; however, it is the most common cause of all deaths from skin cancers in Japan.^2^ According to data from the hospital‐based cancer registries and nationwide statistical surveys from Japan, the reported prevalence was 47.5% for nonacral cutaneous melanoma and 33.0% for acral lentiginous melanoma (ALM), followed by 14.8% for mucosal melanoma, 2.9% for uveal melanoma, and 1.8% for unknown.^2^, ^3^, ^4^ The prevalence of melanoma is different by histological subtypes, with that of ALM and mucosal melanoma being higher in the Asian population (including Japan) compared with the Western/Caucasian population.^3^, ^5^, ^6^, ^7^, ^8^ According to the new classification based on cumulative sun damage (CSD), anatomic location, and genetic abnormality, the incidence of ALM and mucosal melanoma is relatively higher compared with that of non‐CSD, CSD, and uveal melanoma in the non‐White population. The new classification also considers ALM as both site and biologically specific.^8^, ^9^, ^10^, ^11^, ^12^ Furthermore, the incidence of B‐Raf proto‐oncogene (*BRAF*) mutation is low in both acral and mucosal melanoma, especially compared with melanomas on the skin without CSD.^9^, ^11^, ^13^ Compared with other cutaneous melanomas, this lower mutation burden in ALM and mucosal melanomas^11^, ^14^ could potentially explain their poor response to anti‐programmed cell death protein 1 (anti‐PD‐1) therapy.^15^

With the introduction of immune checkpoint inhibitors and other targeted therapies, systemic therapies for advanced melanoma have drastically improved. After the landmark clinical trial that demonstrated a survival benefit with ipilimumab,^16^ the phase 3 KEYNOTE‐006 study demonstrated further improvement in the overall outcome with pembrolizumab versus ipilimumab,^17^ which continued over 5 years of follow‐up.^18^ Furthermore, the phase 3 CheckMate 067 study also showed a sustained survival benefit with first‐line nivolumab plus ipilimumab or nivolumab alone in patients with advanced melanoma.^19^ In addition, in the real‐world setting in Japan, the prospective, observational, CREATIVE study in 124 patients with advanced melanoma showed a higher response rate with nivolumab as first‐line (23.4%) versus second‐line therapy (8.5%).^20^

For all patients in the first‐ and second‐line setting, the European Society for Medical Oncology (ESMO) Clinical Practice Guidelines for the treatment of metastatic cutaneous melanoma recommend (evidence level: II; recommendation: B) PD‐1‐blocking antibodies, such as pembrolizumab and nivolumab, an anti‐cytotoxic T‐lymphocyte‐associated antigen 4 (CTLA4) antibody, such as ipilimumab, or BRAF/mitogen‐activated protein kinase kinase (MEK) inhibitor combinations for patients with *BRAF*‐mutant melanoma when the primary tumor is screened for detection of *BRAF* V600 mutation.^21^ The 2019 Japanese Dermatological Association Guidelines for cutaneous melanoma recommend pembrolizumab for both treated and untreated advanced melanoma.^22^ Furthermore, both first‐ and second‐line or subsequent therapy recommendations in the National Comprehensive Cancer Network (NCCN) Guidelines for metastatic or unresectable melanoma include pembrolizumab monotherapy (category 1).^23^

This all‐case postmarketing surveillance (PMS) survey was conducted as a condition‐of‐approval study to capture the factors that impact the safety and effectiveness of pembrolizumab monotherapy for the treatment of radically unresectable melanoma in clinical practice in Japan.

## 2 METHODS

### 2.1 Study design, patients, and treatment

This all‐case PMS survey was conducted in accordance with the Japanese regulatory requirements stipulated in Good Post‐marketing Study Practice^24^ at 101 centers between February 15, 2017, and March 3, 2020 (data cutoff), in Japan. Patients who had received at least one dose of pembrolizumab were enrolled both retrospectively and prospectively between February 15, 2017, and March 31, 2018, and observed for 1 year after pembrolizumab initiation (data cutoff: March 3, 2020). Eligible patients were followed up at 1, 3, 6, and 12 months after initiating pembrolizumab treatment. All‐case surveillance was performed using an electronic data capture (EDC; InForm of Japanese Oracle K.K.) system that provided data from relevant study sites.

The protocol for the research project was approved by a suitably constituted Ethics Committee of the institution or was approved according to rules of the institution within which the work was undertaken and it conforms to the provisions of the Declaration of Helsinki. Written informed consent from patients, although not mandatory, was obtained based on the requirements of each individual study site.

All patients with radically unresectable melanoma (American Joint Committee on Cancer [AJCC] staging) who were newly initiated on pembrolizumab for the prescribed indication (per prescribing information) were registered for this PMS survey. The approved dose of pembrolizumab, as described in the prescribing information, was 2 mg/kg of body weight (genetic recombination) as an intravenous drip infusion over 30 min every 3 weeks (q3w). In December 2018, a fixed dose was approved, and subsequently patients were switched to a fixed dose of 200 mg/body.

### 2.2 Safety

The adverse event (AE) observation period was set from the start of study drug administration to 30 days after the final administration date of the study drug. Safety assessments were categorized by treatment‐related AEs (TRAEs), AEs of special interest (AEOSIs) from the Japanese Risk Management Plan (J‐AEOSIs), and J‐AEOSIs related to pembrolizumab. The relationship between AEs and treatment and that between J‐AEOSIs and pembrolizumab was based on physician discretion. J‐AEOSIs as described in the Japanese Risk Management Plan^25^ are presented in Supporting Information Table S1.

### 2.3 Effectiveness

Tumor response (complete response [CR], partial response [PR], stable disease [SD], progressive disease [PD], and disease control rate [DCR]) was evaluated according to the Response Evaluation Criteria in Solid Tumors (RECIST v1.1; translates to Japan Clinical Oncology Group [JCOG] v1.0). The treatment duration was categorized by the tumor response. The objective response rate (ORR; CR or PR) was categorized by patient baseline characteristics, including use of concomitant steroids. Effectiveness, clinical course, and biomarker information were collected at the discretion of the physician in charge based on the information obtained under the conditions of routine medical care.

### 2.4 Statistical analysis

The sample size was planned at 250 patients with radically unresectable malignant melanoma, which was based on the incidence of major adverse reactions, such as hypothyroidism (7.5% [72/954]), hyperthyroidism (3.6% [34/954]), colitis (1.9% [18/954]), severe diarrhea (1.6% [15/954]), and pneumonitis (1.0% [10/954]), in 954 patients from clinical studies outside Japan (KEYNOTE‐002^26^ and KEYNOTE‐006^17^) and from a Japanese clinical study (KEYNOTE‐041).^27^ Assuming that the postmarketing incidence was similar, the number of patients was selected as 250, with at least one patient with the 90% confidence available in the data. The subgroups of malignant melanoma according to the site of occurrence are described as “cutaneous malignant melanoma” for cases with skin involvement and “mucosal malignant melanoma” for cases with mucosal involvement.

The cases with ALM are classified into another subgroup, and the analyzed cases are classified into three groups according to the priority of “mucosal malignant melanoma” > “acral lentiginous melanoma” > “cutaneous malignant melanoma.”

The safety analysis set comprised all enrolled patients after excluding patients who met the following exclusion criteria: patients treated outside the contract/enrollment period, overlapping cases, patients lost to follow‐up after the first treatment administration, patients lacking information on AEs, patients with unknown drug administration status in the case report form (CRF), patients without any background information available, or patients participating in other pembrolizumab clinical trials.

The effectiveness analysis set comprised patients included in the safety analysis set after excluding patients who met the following exclusion criteria: patients with missing or unavailable assessments for efficacy and patients treated with off‐label use of the study drug (unmatched category of radically unresectable malignant melanoma or administration of doses not prescribed in the package insert).

The RECIST assessment set comprised all patients (cutaneous malignant melanoma, mucosal malignant melanoma, and ALM) for whom physicians confirmed use of the RECIST criteria for response assessment and who had available background characteristics. Data were summarized descriptively. Continuous values were summarized as mean, standard deviation, median, and range; discrete values were summarized as number and percentage of patients.

## 3 RESULTS

**FIGURE 1:**
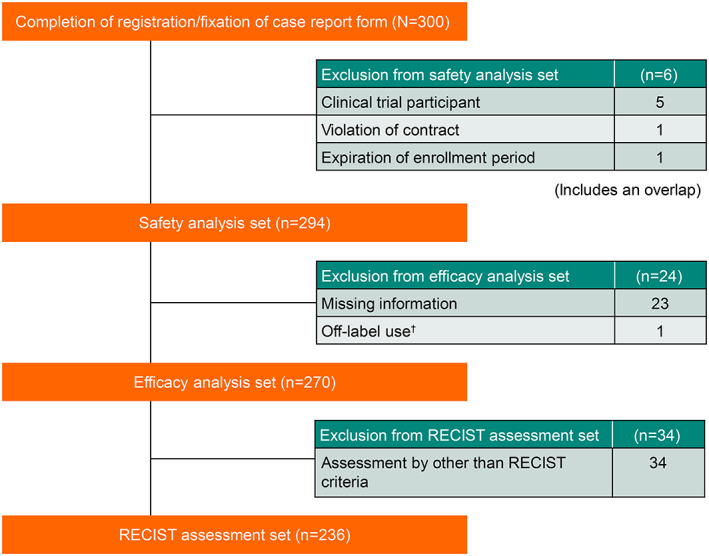
Patient disposition. RECIST, Response Evaluation Criteria in Solid Tumors. ^†^Exclusion criteria based on the Japanese regulatory requirements.

A total of 300 patients were registered, of whom 294 and 270 constituted the safety and effectiveness analysis sets, respectively, while the RECIST assessment set comprised 236 patients. Patient disposition is presented in Figure 1. The median (range) age of the patient population was 70 (22–94) years (>75 years, 36.4%), 56.8% of patients were men, and 38.8% of patients had a disease duration of less than 1 year. The most common type of melanoma was cutaneous (41.5%), followed by mucosal (29.3%: nasal passages/sinus [*n* = 23], rectum/anus [*n* = 18], female genitalia [*n* = 14], and others [*n* = 31]), acral (24.8%), and unknown (4.4%). Most patients had an Eastern Cooperative Oncology Group performance status (ECOG PS) of 0 or 1 (90.5%), with 83.3% in the AJCC stage 3 or 4. Notably, 26.2%, 5.5%, and 1.2% of patients with cutaneous, acral, and mucosal melanomas, respectively, tested positive for *BRAF* mutation (Table 1). A majority (60.4%) of patients received pembrolizumab as first‐line systemic therapy, 21.8% were previously treated with nivolumab, and 29.1% received previous radiotherapy for mucosal malignant melanoma (Table 1).

**TABLE 1:** Baseline characteristics (safety analysis set)

Characteristics, *n* (%)	Total^a^ *N* = 294	Cutaneous *n* = 122 (41.5)	Acral *n* = 73 (24.8)	Mucosal *n* = 86 (29.3)
Age (years)				
Median (range)	70.0 (22–94)	68.5 (22–94)	72.0 (48–92)	70.0 (23–88)
<65 years	97 (33.0)	51 (41.8)	16 (21.9)	25 (29.1)
65–75 years	90 (30.6)	26 (21.3)	26 (35.6)	33 (38.4)
>75 years	107 (36.4)	45 (36.9)	31 (42.5)	28 (32.6)
Sex				
Male	167 (56.8)	74 (60.7)	47 (64.4)	37 (43.0)
Disease duration				
Less than 1 year	114 (38.8)	48 (39.3)	19 (26.0)	39 (45.3)
1–3 years	96 (32.7)	43 (35.2)	32 (43.8)	19 (22.1)
Over 3 years	64 (21.8)	25 (20.5)	21 (28.8)	17 (19.8)
Unknown/no description	20 (6.8)	6 (4.9)	1 (1.4)	11 (12.8)
ECOG PS				
0	199 (67.7)	84 (68.9)	52 (71.2)	58 (67.4)
1	67 (22.8)	29 (23.8)	14 (19.2)	19 (22.1)
2	17 (5.8)	6 (4.9)	4 (5.5)	6 (7.0)
3	7 (2.4)	3 (2.5)	2 (2.7)	1 (1.2)
4	3 (1.0)	—	1 (1.4)	2 (2.3)
Unknown	1 (0.3)	—	—	—
AJCC stage				
I–II	37 (12.6)	14 (11.5)	5 (6.8)	18 (20.9)
III	66 (22.4)	33 (27.0)	22 (30.1)	10 (11.6)
IV	179 (60.9)	73 (59.8)	46 (63.0)	49 (57.0)
Unknown	12 (4.1)	2 (1.6)	—	9 (10.5)
*BRAF* mutation				
(+)	40 (13.6)	32 (26.2)	4 (5.5)	1 (1.2)
(–)	180 (61.2)	64 (52.5)	56 (76.7)	53 (61.6)
Unknown	74 (25.2)	26 (21.3)	13 (17.8)	32 (37.2)
Previous anticancer drug^b^	116 (39.5)	46 (37.7)	37 (50.7)	29 (33.7)
Nivolumab^c^	64 (21.8)	20 (16.4)	21 (28.8)	21 (24.4)
Ipilimumab^c^	41 (13.9)	10 (8.2)	15 (20.5)	14 (16.3)
Interferon	40 (13.6)	17 (13.9)	18 (24.7)	5 (5.8)
Dacarbazine	24 (8.2)	5 (4.1)	9 (12.3)	9 (10.5)
BRAF and/or MEK inhibitors	12 (4.1)	9 (7.4)	2 (2.7)	—
Previous surgery	161 (54.8)	66 (54.1)	50 (68.5)	39 (45.3)
Previous radiotherapy	56 (19.0)	21 (17.2)	7 (9.6)	25 (29.1)
Previous immunotherapy^d^	25 (8.5)	8 (6.6)	11 (15.1)	5 (5.8)

Abbreviations: AJCC, American Joint Committee on Cancer; BRAF, B‐Raf proto‐oncogene; ECOG PS, Eastern Cooperative Oncology Group performance status; MEK, mitogen‐activated protein kinase kinase.

^a^ Total patient number includes 13 patients with unknown tumor types.

^b^ There are overlapping patients due to combination and/or sequential therapies.

^c^ Seven patients received nivolumab and ipilimumab as combination therapy.

^d^ Includes interferons, nivolumab, and ipilimumab.

### 3.1 Treatment exposure

The median (range) frequency of pembrolizumab administration was 6.0 (1.0–22.0) times: 8.0 (1.0–22.0), 7.0 (1.0–21.0), and 4.0 (1.0–20.0) times in the cutaneous, acral, and mucosal melanoma groups, respectively. Median (range) duration of treatment with pembrolizumab was 17.0 (0.1–52.3) weeks: 24.4 (0.1–52.3), 19.1 (0.1–52.3), and 12.0 (0.1–52.3) weeks in the cutaneous, acral, and mucosal melanoma groups, respectively (Table 2). The average (range) number of pembrolizumab doses was 8.2 (1–22) over the 1‐year follow‐up (*n* = 291). Overall, 71% of patients had discontinued pembrolizumab, the most common reason for which was PD (61.1%), followed by AEs (15.9%), death (12.5%), others (6.7%), and loss to follow‐up due to transfer (3.8%).

**TABLE 2:** Treatment profile (safety analysis set)

	Total^a^ *N* = 294	Cutaneous *n* = 122	Acral *n* = 73	Mucosal *n* = 86
Median duration of treatment (range) (weeks)	17.0^b^ (0.1–52.3)	24.4 (0.1–52.3)	19.1 (0.1–52.3)	12.0^c^ (0.1–52.3)
Median number of doses (range)	6.0^b^ (1–22)	8.0 (1–22)	7.0 (1–21)	4.0 (1–20)
Treatment status, *n* (%)				
Continuation	78 (26.5)	37 (30.3)	17 (23.3)	18 (20.9)
Discontinuation	213 (72.4)	85 (69.7)	56 (76.7)	65 (75.6)
Reason for discontinuation, *n* (%)				
Disease progression	131 (61.5)	47 (55.3)	36 (64.3)	44 (51.2)
Adverse event	34 (15.9)	11 (9.0)	13 (17.8)	8 (9.3)
Death	26 (12.2)	13 (10.7)	3 (5.4)	9 (10.5)
Transfer (loss to follow‐up)	8 (3.8)	6 (4.9)	1 (1.4)	1 (1.2)
Others/unknown	14 (6.6)	8 (6.6)	3 (5.4)	3 (3.5)

^a^ Total patient number includes 13 patients with unknown tumor types.

^b^ *n* = 291.

^c^ *n* = 83.

The initial dose of pembrolizumab was 2 mg/kg/dose in all patients (*n* = 294), and some patients were switched to a fixed dose of 200 mg/body after approval in December 2018.

### 3.2 Safety

Overall, 45.2% and 24.8% of patients experienced TRAEs and AEOSIs, respectively. Grade ≥3 TRAEs and AEOSIs were reported in 15.0% and 9.2% of patients, respectively, and were observed with a similar frequency in patients with cutaneous, acral, or mucosal melanomas. No grade 5 J‐AEOSIs were observed (Table 3). Overall, 24.8% and 9.2% of patients experienced any‐grade and grade ≥3 pembrolizumab‐related AEOSIs. Pembrolizumab‐related AEOSIs were reported in a similar proportion of patients with cutaneous (22.1%), acral (26.0%), and mucosal (26.7%) melanomas.

**TABLE 3:** Safety profile (TRAEs and J‐AEOSIs related to pembrolizumab)

	Total *N* = 294
TRAEs, cases (%)	
Any	133 cases (45.2)
Grade ≥3	44 cases^a^ (15.0)
Grade 5	7 cases^a^ (2.4)
Unknown^b^	12 (4.1)
AEs of special interest (J‐AEOSIs), cases (%)	
Any	73 cases^a^ (24.8)
Grade ≥3	27 cases^a^ (9.2)
Grade 5	Not reported
Unknown^b^	3 (1.0)

Abbreviations: AE, adverse event; CTCAE, Common Terminology Criteria for Adverse Events; J‐AEOSI, adverse event of special interest from the Japanese Risk Management Plan; TRAE, treatment‐related adverse event.

^a^ Number of cases with at least one applicable grade event.

^b^ Unknown cases; the CTCAE grade of all TRAEs was not reported.

Grade ≥3 pembrolizumab‐related AEOSIs were reported in a similar proportion of patients with cutaneous (9.0%), acral (8.2%), and mucosal (11.6%) melanomas. Overall, the most common grade ≥3 AEOSIs related to pembrolizumab were endocrine disorders (*n* = 7 [2.4%]; recovered, 1; recovering, 2; not‐recovered, 4) and liver dysfunction (*n* = 7 [2.4%]; recovered, 4; recovering, 3), followed by colitis/severe diarrhea (*n* = 6 [2.0%]; recovered, 3; recovering, 3), interstitial lung disease (ILD; *n* = 3 [1%]; recovered, 1; recovering, 1; not‐recovered, 1), type 1 diabetes mellitus (*n* = 2 [0.7%]; recovering, 1; not‐recovered, 1), renal impairment (*n* = 1 [0.3%]; recovering, 1), myositis/rhabdomyolysis (*n* = 1 [0.3%]; recovering, 1), and uveitis (*n* = 1 [0.3%]; recovered, 1) (Table 4).

**TABLE 4:** AEs of special interest related to pembrolizumab

	Pembrolizumab (*N* = 294) Patients, *n* (%)	
	Any	Grade ≥ 3
Endocrine disorder	29 (9.9)	7 (2.4)
Liver dysfunction	18 (6.1)	7 (2.4)
Interstitial lung disease	15 (5.1)	3 (1.0)
Colitis/severe diarrhea	9 (3.1)	6 (2.0)
Type 1 diabetes mellitus	4 (1.4)	2 (0.7)
Renal impairment	3 (1.0)	1 (0.3)
Uveitis	4 (1.4)	1 (0.3)
Myositis/rhabdomyolysis	1 (0.3)	1 (0.3)
Pancreatitis	1 (0.3)	—
Severe skin reaction	1 (0.3)	—

Abbreviation: AE, adverse event.

Nine grade 5 AEs occurred in seven patients and were not related to pembrolizumab. These were death, malignant neoplasm progression (four cases), hyponatremia/heart failure, and intracranial hemorrhage/seizure.

### 3.3 Effectiveness

The ORR was 16.5% at the 1‐year follow‐up and was twice as high among patients with mucosal melanoma (20%) than among patients with acral melanoma (10%). ORR in patients with cutaneous melanoma was 17.5%. SD and PD were observed in 35.6% and 47.9% of patients, respectively. The DCR was 52.1% at the 1‐year follow‐up and the highest in patients with cutaneous melanoma (57.3%), followed by acral melanoma (51.7%) and mucosal melanoma (43.1%; Table 5). Overall, patients achieving CR or PR received treatment for a longer duration than those with SD and PD (Supporting Information Table S2).

**TABLE 5:** Tumor response by disease types (RECIST assessment set)

Response, *n* (%)	Total^a^ *N* = 236	Cutaneous *n* = 103	Acral *n* = 60	Mucosal *n* = 65
CR	7	4	1	2
PR	32	14	5	11
SD	84	41	25	15
PD	113	44	29	37
ORR	39 (16.5)	18 (17.5)	6 (10.0)	13 (20.0)
DCR	123 (52.1)	59 (57.3)	31 (51.7)	28 (43.1)

Abbreviations: CR, complete response; DCR, disease control rate; ORR, objective response rate; PD, progressive disease; PR, partial response; RECIST, Response Evaluation Criteria in Solid Tumors; SD, stable disease.

^a^ Total patient number includes seven with other and one with unknown tumor types.

When categorized by patient characteristics, the ORR was higher among patients who did not receive previous systemic therapy (20.0%) than among those who received previous systemic therapy (11.4%) across all three melanoma types. Response was only observed in patients with an ECOG PS of 0–1 (17.9%); it was similar among patients with cutaneous (18.8%), mucosal (22.0%), and acral (only ECOG PS of 0; 13.6%) melanomas. A higher ORR was observed in patients who initiated treatment within <1 year of diagnosis (28.1%) versus patients with diagnoses of 1–3 years (10.5%) and >3 years (6.4%). Overall, the ORR increased with increasing duration of treatment up to 52 weeks. Concomitant use of steroids was low and did not affect the ORR (Table 6).

**TABLE 6:** ORR based on patient demographics and characteristics (RECIST assessment set)

*n*/*N* (%)	Total *N* = 236
ORR (%)	39/236 (16.5)
Previous anticancer drug	
−	29/145 (20.0)
+	10/88 (11.4)
Previous RT	
−	30/191 (15.7)
+	9/44 (20.5)
ECOG PS	
0	32/164 (19.5)
1	7/54 (13.0)
≥2	0/17
Time since diagnosis (years)	
<1	27/96 (28.1)
≥1 to <3	8/76 (10.5)
≥3	3/47 (6.4)
*BRAF* mutation	
−	25/152 (16.4)
+	6/33 (18.2)
Duration of treatment with pembrolizumab (weeks)	
0–4	0/34 (0.0)
4–12	2/48 (4.2)
12–26	6/53 (11.3)
26–52	13/58 (22.4)
>52	18/42 (42.9)
Concomitant steroid use	
−	32/187 (17.1)
+	7/44 (15.9)

Abbreviations: ECOG PS, Eastern Cooperative Oncology Group performance status; ORR, objective response rate; RECIST, Response Evaluation Criteria in Solid Tumors; RT, radiotherapy.

## 4 DISCUSSION

This is the first all‐case PMS survey in the immune checkpoint inhibitor era that reflects the real‐world safety and effectiveness of pembrolizumab monotherapy for the treatment of radically unresectable melanoma in Japan. Since the product has been studied in only a limited number of patients in clinical studies in Japan, it required the condition of approval to conduct a drug‐use results survey involving all patients treated with the product after the market launch until data from a certain number of patients had been gathered to grasp the characteristics of treated patients and ensure appropriate use of the product.^28^ In this survey, all‐patient surveillance was started on February 15, 2017, and a total of 300 patients were enrolled. Of 294 patients in the safety analysis set, the incidence of an adverse drug reaction was 45.2% (133 of 294 patients). Based on the results of this survey, no new safety concerns were identified for the drug's single‐agent therapy for unresectable malignant melanoma in Japan. Regarding other safety specifications, such as the occurrence of an adverse drug reaction, there were no results requiring any changes in the risk–benefit profile of pembrolizumab from this PMS survey.

The safety of pembrolizumab has been demonstrated in phase 3 trials for numerous tumor types,^29^, ^30^, ^31^ including melanoma.^17^, ^18^, ^32^, ^33^, ^34^ However, in the current treatment landscape of melanoma in the Japanese population, the safety of pembrolizumab has been demonstrated in a phase 1b trial for melanoma^27^ and phase 3 trials for other tumor types,^35^, ^36^ whereas limited evidence exists from postmarketing and real‐world evidence studies of other immune checkpoint inhibitors such as nivolumab and ipilimumab in the Japanese population with melanoma.^37^, ^38^ The incidence of TRAEs (45.2%; grade ≥3 15.0%) after a median (range) administration period of 17.1 (0.1–52.3) weeks with pembrolizumab treatment in this PMS survey was lower compared with that in the KEYNOTE‐041 (81%; grade ≥3 19.0%) and KEYNOTE‐006 studies (79.5% in the 2‐week dose group and 72.9% in the 3‐week dose group; grade ≥3 13.3% and 10.1%, respectively).^17^, ^27^

The proportion of patients with melanoma subtypes reported in this survey (cutaneous [41.5%], ALM [24.8%], mucosal [29.3%], and others [4.4%]) was not similar to that in the reported registry in Japan.^3^ In addition, the real‐world CREATIVE study from Japan also reported a high prevalence of mucosal melanoma (33.9%), followed by ALM (20.1%), nodular melanoma (15.3%), and superficial spreading melanoma (13.0%),^20^ suggesting that the proportion of mucosal melanoma is increasing as a histological type or as advanced carcinoma requiring pharmacotherapy. A new classification has been shown to categorize melanoma by the cumulative amount of sun exposure (CSD), anatomic location, and genetic abnormality. In this classification, melanoma is categorized into a group with a high cumulative amount of sun exposure (high‐CSD: equivalent to the conventional lentigo maligna type), a group with a low cumulative amount of sun exposure (low‐CSD: equivalent to the conventional superficial spreading type), and other distal extremities (acral: equivalent to the conventional acral lentiginous type), mucosal (mucosal), intraocular (uveal), malignant Spitz tumor, congenital pigmented nevus, and blue nevus.^9^ Due to the relatively high morbidity and the lack of efficacy of pharmacotherapy, we separately analyzed cutaneous melanoma and ALM in this survey.

The proportion of patients with *BRAF* mutations in this survey was similar (13.6%; cutaneous 26.2%, ALM 5.5%, mucosal 1.2%) to that in the CREATIVE study (16.1%; cutaneous 33.0%, ALM 7.0%, mucosal 2.0%) from Japan.^20^ However, the prevalence of these mutations was lower compared with that in the global phase 3 CheckMate 037 (22.0%)^39^ and CheckMate 067 (31.9%) studies^40^ among others.^17^, ^26^, ^32^, ^33^, ^41^ The incidence of *BRAF* mutations in Asian patients with primary melanoma is half of that in White patients^42^; *BRAF* V600E mutations occur at a high rate in low‐CSD melanoma but are less common in other forms, which could also explain the low number of *BRAF* mutations in this survey as more patients with acral subtype were enrolled. In addition, alternative treatment options (MEK/BRAF inhibitors) are prioritized in *BRAF* mutation‐positive patients, which may be a factor limiting the enrollment of patients with *BRAF* mutations in this survey.^23^, ^42^

Of the 236 patients included in the RECIST assessment set, 39 (16.5%) were assessed as responders (CR + PR). The response rate was lower compared with that in the KEYNOTE‐006 development program (pembrolizumab administered once every 2 weeks [q2w] [36.9%] and q3w [36.1%] vs ipilimumab [11.9%]).^17^, ^18^, ^43^ This difference in the response rate could be attributed to nivolumab and ipilimumab (both immune checkpoint inhibitors) that were administered as prior therapies in some patients, suggesting the possibility of including nonresponders who were switched in this survey and the substantially lower incidence of acral melanoma and mucosal melanoma in both small‐ and large‐scale international clinical studies.^13^, ^44^ However, DCR (51.9%) in the RECIST assessment set of this survey was comparable with that in the pembrolizumab phase 3 KEYNOTE‐006 trial (DCR [CR + PR + SD]: 47.6% [q2w] and 46.9% [q3w]).^17^, ^18^, ^43^ The lower ORR observed in this survey (compared with KEYNOTE‐006 studies) could also be attributed to a patient performance status (ECOG PS) of 2/3, as these patients were nonresponders in the real‐world setting in Japan.

Considering melanoma subtypes, the small (*n* = 42) phase 1b KEYNOTE‐041 study of pembrolizumab in Japanese patients with advanced melanoma reported a similar ORR (investigator review) for cutaneous (26.5% [cutaneous + ALM]) and mucosal (37.5%) melanomas with a median follow‐up of 10.3 months.^27^ Although the phase 1b KEYNOTE‐041 study showed some efficacy signal in patients with ALM (*n* = 12) and mucosal melanoma (*n* = 8) subtypes, the patient numbers were limited.^27^

The effectiveness (ORR) of pembrolizumab in this survey was observed in patients with ALM (10.0%) and mucosal melanoma (20.0%). Similarly, the efficacy of anti‐PD‐1 antibody monotherapy in patients with ALM, particularly in those with ungual melanoma, was found to be poor in previous studies.^45^, ^46^ Only three of 67 patients with ALM had ungual melanoma in the effectiveness analysis in this survey. Among these three patients, one was evaluated to have a PR, while the others had SD. The accuracy of ORR evaluation in this survey has limitations for comparison with previous studies due to the limited number of patients included in the RECIST assessment set and the inclusion of patients with a treatment history or poor performance status.

The CREATIVE study from Japan also reported similar investigator‐assessed ORR (17.7%; ALM 16% [4/25]; mucosal 19% [8/42]) and DCR (41.1%; ALM 56% [14/25]; mucosal 38.1% [16/42]) with nivolumab in patients with advanced melanoma compared with this survey.^20^ In the CREATIVE study, mucosal melanoma was the most common clinical subtype (33.9% [42/124]), followed by ALM (20.2% [25/124]). This is in line with previous reports, suggesting that there exists an unmet medical need in the patient population with ALM,^47^, ^48^, ^49^, ^50^, ^51^ especially in the Japanese population.^5^, ^20^

In Asia, including Japan, the prevalence of mucosal melanoma and ALM is high compared with Western countries.^3^, ^5^, ^6^, ^7^, ^8^, ^52^, ^53^ Therefore, when treating melanoma, it is important to understand these geographical differences in the prevalence of melanoma subtypes and consequently their treatment outcome. A post hoc analysis of pembrolizumab‐treated patients with advanced mucosal melanoma in the global KEYNOTE‐001, ‐002, and ‐006 studies showed that only 84 of 1567 (5.0%) patients had mucosal melanoma compared with 29.3% in this survey. In these 84 patients with mucosal melanoma, ORR was 19% and DCR was 31%.^44^ Owing to the rarity of mucosal melanoma, prospective clinical studies assessing the efficacy of systemic therapy have been rare, although a high unmet medical need exists.

### 4.1 Limitations

Study limitations included the following: the observation period was limited to 1 year, the effectiveness measures were based on physicians' assessment without a central review, statistical evaluations were not performed, and data were analyzed descriptively without source data verification.

## 5 CONCLUSIONS

The results of this PMS survey confirm the safety and effectiveness of pembrolizumab for the treatment of radically unresectable melanoma in Japan. The DCR was comparable with that reported in the previous phase 3 trial of pembrolizumab (KEYNOTE‐006),^17^ and pembrolizumab monotherapy was well tolerated with a manageable safety profile in Japanese patients with unresectable melanoma. Overall, no new safety concerns were observed in the real‐world setting over the 1‐year follow‐up period. This is an all‐case PMS survey, and the results represent real‐world observations of pembrolizumab monotherapy in Japan.

## CONFLICT OF INTEREST

NY received lecture fees from Ono and Novartis and research funding and joint research expenses from Ono, BMS, Novartis, and MSD. MO, MH, NT, AS, YI, and SM are employed by and own stock in MSD.

## Supporting information


Table S1–S2

## Data Availability

The data sets analyzed during this PMS survey are not available because data sharing with third parties were not included in the contract with all study sites or patients.
