# Supplementary material for: Real‐world safety and effectiveness of pembrolizumab in Japanese patients with radically unresectable melanoma: An all‐case postmarketing surveillance in Japan
Source: J Dermatol. 2022 Jul 27;49(11):1096–105. doi: 10.1111/1346-8138.16518 (PMC9796869; doi:10.1111/1346-8138.16518)
Supplement: Supplementary file 1 — Table S1–S2 [file JDE-49-1096-s001.docx]

**Supporting information**

**Supporting Table 1.** J-AEOSIs as described in the Japanese Risk Management Plan

| J-AEOSIs as described in the Japanese Risk Management Plan^15^ include the following specific preferred terms for each AEOSI category: ILD; colitis/severe diarrhea; hepatic function disorder; renal function disorder (tubulointerstitial nephritis, etc.); endocrinological disorder (pituitary function disorder, thyroid function disorder, adrenal function disorder); type 1 diabetes mellitus; infusion reaction; pembrolizumab administration in patients with organ transplant history, including a medical history of hematopoietic stem cell transplant; uveitis; myositis/rhabdomyolysis; pancreatitis; nerve disorders (Guillain-Barré syndrome, etc.); severe skin disorder (Stevens-Johnson syndrome, erythema multiforme, pemphigoid, etc.); encephalitis/meningitis; myasthenia gravis; myocarditis; immune thrombocytopenic purpura; hemolytic anemia; and/or aplasia pure red cell. |
| --- |

AEOSI, adverse event of special interest; ILD, interstitial lung disease; J-AEOSI, AEOSI from the Japanese Risk Management Plan.

**Supporting Table 2.** Treatment duration categorized by tumor response (RECIST effectiveness set)

| **Total** | | | | | | |
| --- | --- | --- | --- | --- | --- | --- |
| **Tumor response** | **Number of cases** | **Average value (days)** | **Standard deviation** | **Minimum value (days)** | **Median (days)** | **Maximum value (days)** |
| CR | 7 | 245.9 | 113.8 | 61 | 301.0 | 358 |
| PR | 32 | 234.8 | 110.0 | 43 | 230.5 | 365 |
| SD | 84 | 175.0 | 99.5 | 14 | 163.5 | 365 |
| PD | 113 | 97.3 | 78.7 | 4 | 69.0 | 322 |
| **Cutaneous malignant melanoma** | | | | | | |
| CR | 4 | 329.5 | 32.3 | 301 | 329.5 | 358 |
| PR | 14 | 245.6 | 109.4 | 43 | 252.0 | 365 |
| SD | 41 | 181.7 | 102.9 | 14 | 166.0 | 354 |
| PD | 44 | 100.0 | 74.2 | 6 | 83.5 | 322 |
| **Acral melanoma** | | | | | | |
| CR | 1 | 147.0 | - | 147 | 147.0 | 147 |
| PR | 5 | 196.2 | 106.5 | 90 | 191.0 | 336 |
| SD | 25 | 197.4 | 102.7 | 42 | 188.0 | 365 |
| PD | 29 | 103.7 | 82.6 | 7 | 69.0 | 299 |
| **Mucosal malignant melanoma** | | | | | | |
| CR | 2 | 128.0 | 94.8 | 61 | 128.0 | 195 |
| PR | 11 | 229.5 | 122.2 | 49 | 188.0 | 364 |
| SD | 15 | 124.7 | 69.6 | 21 | 118.0 | 245 |
| PD | 37 | 91.8 | 83.6 | 4 | 61.0 | 321 |

CR, complete response; PD, progressive disease; PR, partial response; RECIST, Response Evaluation Criteria in Solid Tumors; SD, stable disease.
